# Correlation Analysis on Anatomical Variants of Accessory Foramina in the Sphenoid Bone for Oncological Surgery

**DOI:** 10.3390/cancers15225341

**Published:** 2023-11-09

**Authors:** Andrea Palamenghi, Michaela Cellina, Maurizio Cè, Annalisa Cappella, Chiarella Sforza, Daniele Gibelli

**Affiliations:** 1 Dipartimento di Scienze Biomediche per la Salute, Università degli Studi di Milano, Via L. Mangiagalli, 31, 20133 Milan, Italy; 2 Reparto di Radiologia, Ospedale Fatebenefratelli, ASST Fatebenefratelli Sacco, Piazza Principessa Clotilde, 3, 20121 Milan, Italy; 3 Scuola di Specializzazione in Radiodiagnostica, Università degli Studi di Milano, Via Festa del Perdono, 7, 20122 Milan, Italy; 4 U.O. Laboratorio di Morfologia Umana Applicata, IRCCS Policlinico San Donato, 20097 San Donato Milanese, Italy

**Keywords:** sphenoid bone, foramen meningo-orbitale, foramen of Vesalius, canaliculus innominatus, palatovaginal canal, CT-scan

## Abstract

**Simple Summary:**

Anatomical variants are traits that differ between individuals. The sphenoid bone has numerous variant features, such as foramina and canals, that may be involved in surgical procedures. This study on CT-scan images describes the foramen meningo-orbitale, the foramen of Vesalius, the canaliculus innominatus and the palatovaginal canal and their relationships. For the first time, a correlation between the foramen of Vesalius and the canaliculus innominatus was found. This topic is especially important in cranial base surgical procedures that may involve tumor treatment.

**Abstract:**

The sphenoid bone presents several anatomical variations, including accessory foramina, such as the foramen meningo-orbitale, the foramen of Vesalius, the canaliculus innominatus and the palatovaginal canal, which may be involved in tumor invasion or surgery of surrounding structures. Therefore, clinicians and surgeons have to consider these variants when planning surgical interventions of the cranial base. The prevalence of each variant is reported in the published literature, but very little information is available on the possible correlation among different variants. Here, 300 CT scans of patients (equally divided among males and females) were retrospectively assessed to investigate the presence of the foramen meningo-orbitale, the foramen of Vesalius, the canaliculus innominatus and the palatovaginal canal. Possible differences in the prevalence of each accessory foramen according to sex were assessed, as well as possible correlations among different variants through the Chi-square test (*p* < 0.01). Overall, the prevalence of the foramen meningo-orbitale, the foramen of Vesalius, the canaliculus innominatus and the palatovaginal canal was 30.7%, 67.7%, 14.0% and 35.3%, respectively, without any difference according to sex (*p* > 0.01). A significant positive correlation was found between the foramen of Vesalius and canaliculus innominatus, both in males and in females (*p* < 0.01). In detail, subjects with canaliculus innominatus in 85.7–100.0% of cases also showed the foramen of Vesalius, independently from sex and side. The present study provided novel data about the prevalence of four accessory foramina of the sphenoid bone in an Italian population, and a correlation between the foramen of Vesalius and the canaliculus innominatus was found for the first time. As these accessory foramina host neurovascular structures, the results of this study are thus useful for appropriate planning surgical procedures that are tailored to the anatomical configuration of the patient and for improving techniques to avoid accidental injuries in cranial base surgery. Knowledge of the topography, frequencies and the presence/absence of these additional foramina are pivotal for a successful procedure. Clinicians and surgeons may benefit from these novel data for appropriate recognition of the variants, decision-making, pre-operative and treatment planning, improvement of the procedures, screening of patients and prevention of misdiagnosis.

## 1. Introduction

In recent decades, surgery of the cranial base has seen a progressive increase in fields of application and treated oncological pathologies [1]. Therefore, precise knowledge of the anatomy of the cranial base is crucial to improve the reliability of surgical procedures, including tumor resection and treatment [1,2,3]. However, several anatomical variants involve the cranial base, the most important of which is the presence of accessory canals, usually within the sphenoid bone and containing vascular or nervous structures. The sphenoid bone is a complex osteological structure that acts as a keystone in the cranial architecture, providing structural integrity to the skull and creating passages and spaces for vital structures. The larger part of the bone is located in the middle cranial fossa, although some structures determine the posterior part of the anterior cranial fossa. At its center is the body carrying paired paranasal sinuses (i.e., sphenoidal sinuses) and the sella turcica with the hypophyseal fossa for the pituitary gland. Anterior to the body are two lesser wings which enclose the optical foramina. Two greater wings extend laterally from the body carrying three main foramina (the foramen rotundum, the foramen ovale and the foramen spinosum), which host vital neurovascular structures (e.g., branches of the trigeminal nerve, arteries and veins). On the ectocranial surface of the body, two medial and two lateral pterygoid processes extend inferiorly for the attachment of masticatory muscles and of other structures of the neck [4].

The sphenoid bone may present several other accessory and variable canals, among which are the foramen meningo-orbitale, the foramen of Vesalius, the canaliculus innominatus, and the palatovaginal canal.

**Figure 1:**
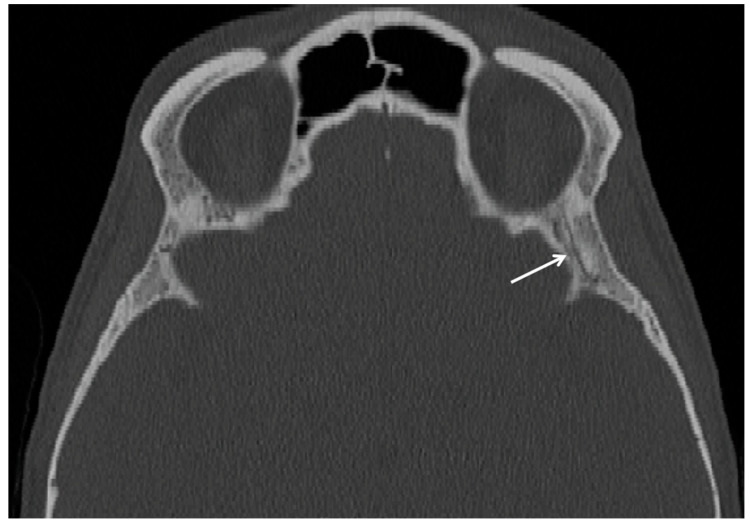
Tranverse CT scan of an individual with the left foramen meningo-orbitale (white arrow).

The foramen meningo-orbitale (also known as foramen of Hyrtl, lacrimal foramen or cranio-orbital foramen) is located within the greater wing, lateral to the superior orbital fissure [5]. It usually contains the orbital branch of the anterior division of the middle meningeal artery and the lacrimal branch of the ophthalmic artery (Figure 1), and occurs in 6.0–82.9% of crania, according to ethnicity [6,7,8]. The foramen meningo-orbitale may be misinterpreted as a cranial fracture, and its recognition is critical to avoid potential iatrogenic vascular injuries in lateral orbital wall surgical interventions [9,10,11,12].

**Figure 2:**
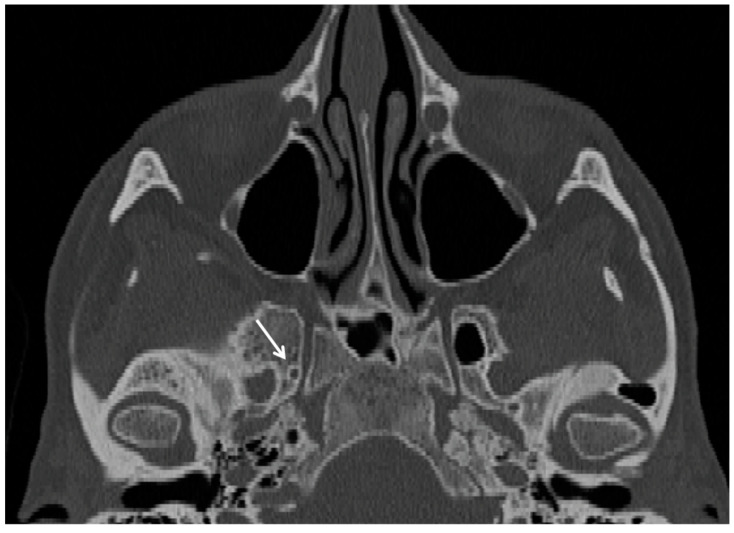
Tranverse CT scan of an individual with the right foramen of Vesalius (white arrow).

The foramen of Vesalius is another anatomical variant of the sphenoid greater foramen, located postero-medially to the foramen rotundum and antero-medially to the foramen ovale (Figure 2). It usually hosts an anastomotic vein between the cavernous sinus and the pterygoid plexus [5] and is reported in 16.0–40.0% of crania [13,14]. The foramen of Vesalius represents a possible risk of vascular lesions in case of surgical interventions of the cranial base; moreover, this variant may transmit through the cranial base several pathological conditions, including nasopharyngeal tumors [15].

**Figure 3:**
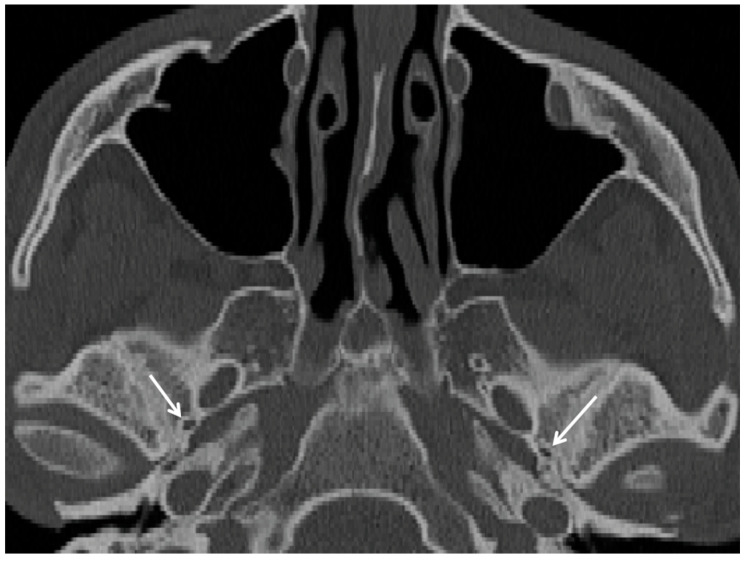
Tranverse CT scan of an individual with the bilateral canaliculus innominatus (white arrows).

The canaliculus innominatus (also known as foramen petrosum or foramen of Arnold) is located in the sphenoid greater wing between the foramen spinosum and the foramen ovale (Figure 3). It surrounds the lesser superficial petrosal nerve and can be found in 16.0% circa of crania [16].

**Figure 4:**
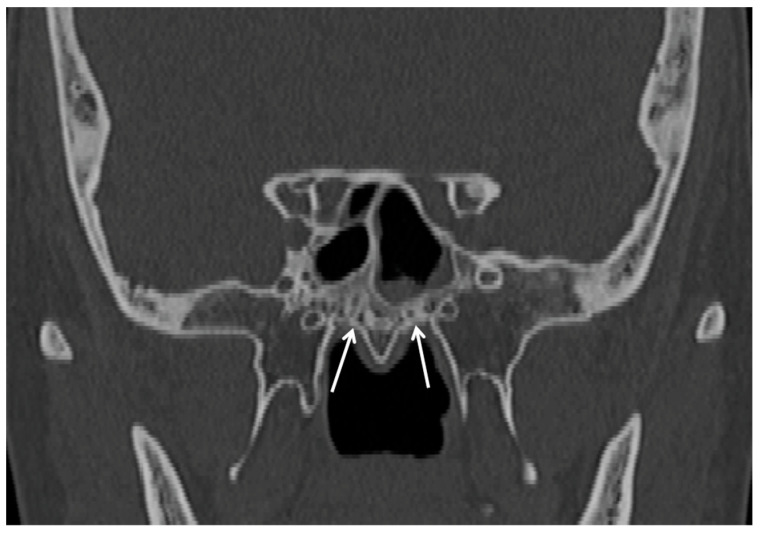
Coronal CT scan of an individual with bilateral palatovaginal canal (white arrows).

The palatovaginal canal (Figure 4), also called the palatopshenoidal canal, is a channel running between the sphenoid process of the palatine bone and the antero-inferior wall of the sphenoid sinus, infero-medially to the posterior wall of the pterygopalatine fossa [17]. It can be found in 27.0–72.5% of crania according to ethnicity [18,19], and hosts the pterygovaginal artery (branch of the internal maxillary artery) and the pharyngeal nerve from the pterygopalatine ganglion to the pharyngeal orifice of the auditory tube [18]. The pterygopalatine artery may anastomose with the ascending pharyngeal and ascending palatine arteries; therefore, it may represent a cause of epistaxis refractory to surgical treatment [19,20]. This canal was recently investigated as a possible origin site of nasopharyngeal tumors [20,21].

Several studies have investigated the aforementioned variants over time, usually reporting their prevalence in different population groups. However, thus far, no research focusing on a possible correlation between the different variants has been produced. The present study aims at verifying the possible coexistence of different types of accessory canals in the sphenoid bone. By expanding the knowledge on frequencies of the abovementioned variants in a specific population, the results of this study will contribute to raise surgeons’ awareness towards these important variants of the cranial base, including surgical treatment of neoplasms [22].

## 2. Materials and Methods

Three hundred maxillofacial CT scans of patients (equally divided among males and females) were retrospectively assessed from a CT-scan database of the FatebeneFratelli hospital in Milan, Italy. Age of males and females was 49.0 ± 19.9 years and 52.6 ± 21.0 years, respectively. Differences of age according to sex was assessed through Student’s *t*-test (*p* < 0.05).

The CT scans were performed between 2015 and 2020 through a second generation dual-source scanner. Somatom Definition Flash (Siemens. Forchheim. Germany) with the following parameters of acquisition: kV: 120, mAs: 320, collimation: 40 × 0.6 mm, tube rotation: 1 sec; reconstruction thickness: 1 mm; reconstruction filters: H21s smooth for soft tissues and H60 sharp for bone was used. CT scans were requested for screening of cranial fractures in trauma, sinusitis, and neurological symptoms. Subjects affected by traumatic injuries and congenital or acquired cranial deformation and pathologies involving the cranial base were excluded from the study. The study follows international guidelines (Helsinki Declaration) and was approved by the local ethical committee Sacco Area 1 (7331/2019).

The possible presence of foramen meningo-orbitale, foramen of Vesalius, canaliculus innominatus and palatovaginal canal was assessed in each CT scan. The evaluations were jointly performed by two authors, both with more than 10 years of experience in the assessment of radiological examinations and anatomical variants.

Possible differences in the prevalence of each accessory foramen according to sex were assessed through the Chi-square test (*p* < 0.01). Possible coexistence among different accessory canals was assessed through the Chi-square test, applied to two variants at a time in males and females, separately (*p* < 0.01).

## 3. Results

No significant difference in age according to sex was found (*p* > 0.05).

The average prevalences of the different accessory foramina of the sphenoid bone are shown in Table 1: foramen meningo-orbitale was found in 24.7% of males, and 36.7% of females; foramen of Vesalius was reported in 70.0% of males and 65.3% of females; canaliculus innominatus occurred in 14.7% of males, and 13.3% of females; palatovaginal canal was shown in 39.3% males and 31.3% females.

No accessory canal of the sphenoid bone showed a significant difference according to sex (Table 2, *p* > 0.01).

The results on the correlation among different accessory canals are reported in Table 3 and Table 4; a significant positive correlation was found between the foramen of Vesalius and the canaliculus innominatus, both in males and in females (*p* < 0.01).

Table 5 and Table 6 report the number of subjects with coexistence of the four different variants: almost all subjects with canaliculus innominatus also showed the foramen of Vesalius, independently from sex and side, with prevalence on average between 85.7% and 100.0%.

## 4. Discussion

Awareness and extensive knowledge of anatomical variants of the cranial base are paramount for surgical practice, especially in the treatment of neoplasms involving cranial structures [21,22,23,24,25,26]. In fact, some accessory canals, usually located in the sphenoid bone, host vascular and nervous structures which may be accidentally injured during surgical procedures, or they may represent transmission pathways for the progression of pathological conditions [4,20]. Among the critical structures that may be observed in cranial base surgery are the meningo-orbital foramen, the foramen of Vesalius, the canaliculus innominatus and the palatovaginal canal.

The most documented and discussed anatomical variant is the meningo-orbital foramen, especially for what concerns differences in prevalence according to ethnicity. The population group considered here presented the variant on average in 30.7% of cases, close to prevalences reported for a Polish (43.0%) [9] and a Serbian group (43.3%) [27]. However, a higher prevalence was reported in an American population (50.0%) [6], an Italian (54%) [12] and a Polish (69.9%) [8]. Considerably higher rates were observed in in Scottish (73.0%) [28], Indian (80.4%) [7] and Turkish (82.9%) [29] groups. On the other side, a lower prevalence (6.0%) was reported in Brazilians [30]. Discordances in the literature also concern the sexual dimorphism of foramen meningo-orbitale; the present study did not find significant differences according to sex, similarly to Tomaszewska and Zelazniewicz [8]. On the other side, Kwiatkowski et al. [9] maintain that the variant is more frequent in females than in males, whereas Krishnamurthy et al. [7] reported that it is more observed in males than in females. Clinical relevance of the meningo-orbital foramen mainly concerns the passage of the meningo-lacrimal artery, which demands caution in lateral or transcranial surgical procedures to treat lesions of the orbit [12]. Injuries to the structures within the meningo-orbital foramen may jeopardize the operation with collateral damages to structures in the superior orbital fissure with possible complications, such as visual disturbances, diplopia, orbital dystopia, enophthalmos, and facial asymmetry [12,31,32].

In this study, the foramen of Vesalius was observed in 67.7% of the sample, which is the highest one recorded in the literature. A considerably lower prevalence was reported in Turkish (41.1%) [26], Indians (49.1%) [33] a Brazilian population (33.8–40.0%) [14,34], a Polish (22.0%) [35] and a Japanese population (21.8%) [36]. As for sexual dimorphism, the present article did not find any significant difference according to sex, whereas the literature reports that the foramen of Vesalius is more frequent in males [13]. Presence and topographic anatomy of the foramen of Vesalius, and the structures involved, may play an important role in nasopharyngeal tumor spread [13], and the surgical treatment of the middle cranial fossa for the biopsy of lesions of the cavernous sinus [33,37] and transcutaneous intervention for trigeminal neuralgia [38].

Less epidemiological data are available about the other two variants. For the canaliculus innominatus, a frequency of 14.0% was observed, similar to data from an American group in an (16.0%) [16] and a Turkish population (17.1%) [26]. Proper identification of this small canal is relevant to avoid confusion between greater and lesser petrosal nerves in surgery of the middle fossa [39].

For the palatovaginal canal, a prevalence of 35.3% was found, closer to data reported in an American population (27.0%) [18], but lower than in a Chinese one (72.5%) [19]. Therefore, at least for the palatovaginal canal, ethnic variability cannot be excluded. The arteries passing through the palatovaginal canal may be involved in surgical procedures of the surrounding structures, e.g., vidian neurectomy [40], anterior sphenoidotomy [41] or in case of severe epistaxis [20,41]. Identification of the palatovaginal canal and the related structures may be important in the detection of tumors advancing from the nasopharynx to the pterygopalatine fossa [18,20], or in the lateral mobilization of soft tissue in the pterygopalatine fossa in nasopharyngeal carcinomas [20,42]. Moreover, recent evidence suggests that the palatovaginal canal may be the onset site for angiofibroma in juvenile individuals [21]. No data about possible differences in prevalence of the two variants according to sex are available; the present article did not find appreciable differences in prevalence between males and females.

All the above-mentioned variants have two features in common: they involve the formation of accessory canals within the cranial base, with the passage of vascular or nervous branches, and they are all located within the sphenoid bone, especially in correspondence with the greater wings (foramen meningo-orbitale, foramen of Vesalius, canaliculus innominatus) and the body (palatovaginal canal). Their origin, in most cases, is unknown, but one may wonder if a correlation among these different variants exists. Yet, the literature has always focused on the mere report of the prevalence of each anatomical variant, without attempting to analyze their possible coexistence.

The present article aimed to verify the possible coexistence of the abovementioned anatomical variants; results showed that the four variants are largely independent from each other, with the only exception of the relationship between canaliculus innominatus and foramen of Vesalius. In detail, almost all subjects with canaliculus innominatus presented the foramen of Vesalius as well, both in males and females, independently from the side. In other words, subjects showing a canaliculus innominatus between foramen spinosum and foramen ovale almost always have, on the same side, a foramen of Vesalius between foramen rotundum and foramen ovale. To the best of our knowledge, this is novel information with potential relevance for surgical interventions of the cranial base. For example, the presence of foramen of Vesalius represents a possible risk of iatrogenic injuries of vascular structures passing through, and, in detail, the anastomotic vein between the cavernous sinus and the pterygoid plexus [5].

Limitations to this study should be acknowledged. This study was performed on 300 CT scans from a set of an Italian hospital, thus the evidence brought here should be further verified in other population groups. In detail, the possible existence of the correlation between foramen of Vesalius and canaliculus innominatus should be investigated in other groups. However, few epidemiological data are available for the canaliculus innominatus, and a wide variability according to ethnicity was already reported for the foramen of Vesalius. This correlation has not been clarified yet, but it may involve a possible common embryologic origin, which is even more likely if one considers the similar position of both variants (close to foramen rotundum and foramen ovale). Further studies are needed to expand our knowledge about the development of these variants and the possible reasons that may explain this correlation.

## 5. Conclusions

This paper collected novel data about frequencies and relationships among different anatomical variants involving accessory canals in the sphenoid bone. The foramen of Vesalius was the most frequent, followed by the foramen meningo-orbitale and the palatovaginal canal. Low frequencies of the canaliculus innominatus were recorded. The data are in line with previous studies, although this study reported the highest frequency of foramen of Vesalius based on the available literature. Moreover, for the first time, a significant positive correlation was found between the foramen of Vesalius and canaliculus innominatus, both in males and in females. Further studies may clarify the possible causes of this coexistence and verify the same correlation in other population groups.

The variable foramina and canals considered here, and the related structures passing through them and communicating with the whole cranium, may be heavily involved in the spread and advancement of neoplasms (e.g., nasopharyngeal tumors, angiofibroma) and in the surgical treatment of neuralgia, neurectomy, tumor resection, epistaxis, transcranial and percutaneous lesion treatment, and biopsy. Unexpected damages to the structures in these variants may lead to severe complications that extend the time for the surgical procedure and the recovery of the patient. Knowledge of the topography, frequencies and the presence/absence of these additional foramina are pivotal for a successful procedure. Clinicians and surgeons may benefit from these novel data for appropriate recognition of the variants, decision-making, pre-operative and treatment planning, improvement of the procedures, screening of patients and prevention of misdiagnosis.

## Figures and Tables

**Table 1:** Prevalence (in percentage) of sphenoid foramina in males and females.

		Foramen Meningo-Orbitale (%)	Foramen of Vesalius (%)	Canalicus Innominatus (%)	Palatovaginal Canal (%)
Males	Right	20.0	60.7	11.3	39.3
	Left	18.7	63.3	10.0	37.3
	Total	24.7	70.0	14.7	39.3
Females	Right	32.0	52.0	9.3	31.3
	Left	24.7	60.7	7.3	30.0
	Total	36.7	65.3	13.3	31.3

**Table 2:** Differences in prevalence of sphenoid foramina according to sex (Chi-square test, *p* < 0.01). In brackets are the *p*-values.

	Right Side	Left Side
Foramen meningo-orbitale	5.613 (0.018)	1.591 (0.207)
Foramen of Vesalius	2.290 (0.130)	0.226 (0.634)
Canalicus innominatus	0.324 (0.569)	0.674 (0.412)
Palatovaginal canal	1.472 (0.225)	2.499 (0.114)

**Table 3:** Results of Chi-square test for testing correlation among different anatomical variants of sphenoid foramina in males. In brackets are the *p*-values. *: *p* < 0.01.

	Right Side	Foramen Meningo-Orbitale	Foramen of Vesalius	Canalicus Innominatus	Palatovaginal Canal
Left Side					
Foramen meningo-orbitale			0.130 (0.114)	0.032 (0.702)	0.143 (0.080)
Foramen of Vesalius		0.116 (0.158)		0.245 * (0.003)	0.145 (0.076)
Canalicus innominatus		−0.046 (0.579)	0.254 * (0.002)		0.143 (0.082)
Palatovaginal canal		0.196 (0.016)	0.130 (0.114)	0.156 (0.056)	

**Table 4:** Results of Chi-square test for testing correlation among different anatomical variants of sphenoid foramina in females. In brackets are the *p* values. *: *p* < 0.01.

	Right Side	Foramen Meningo-Orbitale	Foramen of Vesalius	Canalicus Innominatus	Palatovaginal Canal
Left Side					
Foramen meningo-orbitale			0.116 (0.159)	0.026 (0.749)	0.122 (0.137)
Foramen of Vesalius		0.018 (0.832)		0.217 * (0.008)	0.189 (0.021)
Canalicus innominatus		0.076 (0.353)	0.227 * (0.005)		−0.019 (0.817)
Palatovaginal canal		0.165 (0.043)	0.110 (0.179)	−0.017 (0.839)	

**Table 5:** Number of subjects with coexistence of different accessory canals of sphenoid bone (two variants at a time) in males.

	Right Side	Foramen Meningo-Orbitale (n° 30)	Foramen of Vesalius (n° 91)	Canalicus Innominatus (n° 17)	Palatovaginal Canal (n° 59)	
Left Side						
**Foramen meningo-orbitale (n° 28)**			21	4	16	Foramen meningo-orbitale (n° 30)
**Foramen of Vesalius (n° 95)**		21		16	41	Foramen of Vesalius (n° 91)
**Canalicus innominatus** **(n° 15)**		2	15		10	Canalicus innominatus (n° 17)
**Palatovaginal canal (n° 56)**		16	40	9		Palatovaginal canal (n° 59)
		Foramen meningo-orbitale (n° 28)	Foramen of Vesalius (n° 95)	Canalicus innominatus (n° 15)	Palatovaginal canal (n° 56)	

**Table 6:** Number of subjects with the coexistence of different accessory canals of sphenoid bone (two variants at a time) in females.

	Right Side	Foramen Meningo-Orbitale (n° 48)	Foramen of Vesalius (n° 78)	Canalicus Innominatus (n° 14)	Palatovaginal Canal (n° 47)	
Left Side						
Foramen meningo-orbitale (n° 37)			29	5	19	Foramen meningo-orbitale (n° 48)
Foramen of Vesalius (n° 91)		23		12	31	Foramen of Vesalius (n° 78)
Canalicus innominatus (n° 11)		4	11		4	Canalicus innominatus (n° 14)
Palatovaginal canal (n° 45)		16	31	3		Palatovaginal canal (n° 47)
		Foramen meningo-orbitale (n° 37)	Foramen of Vesalius (n° 91)	Canalicus innominatus (n° 11)	Palatovaginal canal (n° 45)	

## Data Availability

Data are contained within the article.
